# “Working on microbes in everything”: an interview with Denise Akob on the natural microbiome and sustainability

**DOI:** 10.1038/s42003-021-02300-0

**Published:** 2021-06-25

## Abstract

Denise Akob is a Research Microbiologist for the United State Geological Survey (USGS), based at the Geology, Energy & Minerals Science Center in Reston, VA. Dr. Akob received her Ph.D. in 2008 from Florida State University and completed a postdoctoral research fellowship at Friedrich Schiller University Jena before starting her independent research career with the USGS in 2012. In this Q&A, Dr. Akob tells us about her current work, experiences in federal research, and the best bacterial taxa.

**Figure:**
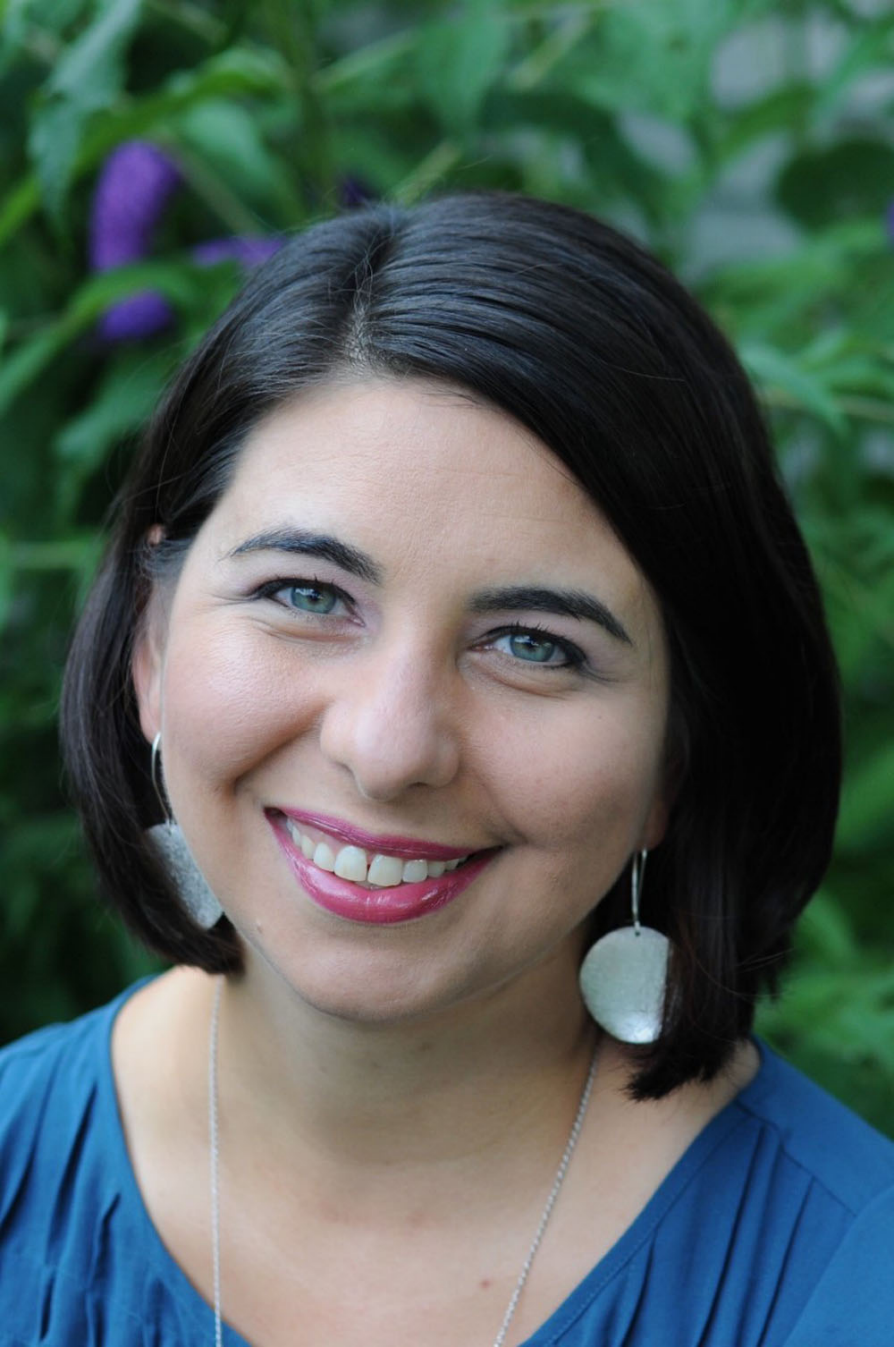
Denise Akob

Can you tell us about your research interests?

I am a highly interdisciplinary scientist with a background in both Life and Earth Sciences and my research sits at the interface between microbiology, geochemistry, and geology. The aim of my research is to understand interactions between microbes and their environment in order to help solve anthropogenic issues. I’ve been described as working on “microbes in everything”, which is mostly true! However, I don’t work on pathogens. The focus of my work is on naturally-occurring microbiomes in soils, sediments, water, and rocks. My team, the Reston Microbiology Lab, works to understand how these environmental microbes degrade contaminants, act as indicators of ecosystem health, affect water quality, and contribute to energy production. A major focus of our research is investigating the effects of energy development and contaminant releases on environmental health, specifically to understand how microorganisms can be tracers of impacts or mitigate contaminants via biodegradation or mineral interactions. Right now, we are studying crude oil spills, oil and gas wastewater releases, and sites contaminated with per- and polyfluoroalkyl substances (PFAS).

What inspired you to pursue a career in federal research, and what do you think are the key differences from an academic research environment?

Great question — interestingly I never expected to have a career in the federal government — I always expected to continue in the university setting as a professor, as I enjoy mentoring students, doing research, and teaching, and didn’t know much about non-academic jobs. The U.S. Geological Survey (USGS) position was appealing to me because it is 100% research and allows me to focus on using interdisciplinary science to solve problems. The key differences I see between a federal position and academia are the funding model and the links to stakeholders. As a taxpayer-funded organization we’re closely tied to the needs of the public and our stakeholders, including land managers, private citizens, and resource managers. While the USGS is a non-regulatory agency, our funding comes directly from Congress and science priorities are set by the administration. Working under that framework can be a challenge but it is truly satisfying to do work that is critical for understanding, utilizing, and protecting our Nation’s resources. One aspect of my job that I really enjoy is working directly with stakeholders and knowing who uses the information from my research. I like seeing that my science can help inform stakeholder decisions.

How has the shutdown caused by the current pandemic affected your lab?

As a government scientist, shutdowns are unfortunately part of the job – in the 8 years I’ve been with the USGS we’ve had 3 federal government shutdowns due to lapses in appropriations. In 2019, we were shut down for 5 weeks, so in some ways we’re experienced with suddenly closing our doors and pausing our science. However, we weren’t prepared for the mental toll of a pandemic or finding office spaces in our homes overnight. We also never expected that a pandemic would last longer than a government shutdown! The most amazing thing was seeing the resilience of my colleagues and watching how we all worked quickly to redefine what work looks like and stay connected via video calls. Most members of my team primarily conduct lab work, so without a lab we had to rethink their day-to-day tasks and change priorities. This renewed our focus on data analysis and interpretation and writing papers on existing data instead of starting new experiments. However, we have a lot of backlogged samples that need to be analyzed and now that we’re able to be in the building a bit more we’re working hard to catch up. Overall, the pandemic has been productive for us as we’ve released a number of publications.

This year’s theme for World Microbiome Day is “sustainability.” Why do you think microbiome and geomicrobiology research are important, and how do they contribute to sustainability efforts?

When I think of sustainability, I think immediately of the impact humans are having on the environment and the need to come up with strategies to balance human needs with the natural world. Since microbes are everywhere and form the base of the food chain, I feel that microbiome and geomicrobiology research is really poised to tackle sustainability. Research to characterize microbiomes is expanding in all kinds of environments from human health to agriculture to waste disposal to the subsurface. I see the field shifting from asking questions about individual organisms to communities (whole microbiomes) and thinking about how to favor the natural ecological interactions that allowed for microbiomes to evolve and be sustained over millennia. We’re seeing a bit of that now with agricultural research shifting from focusing on controlling a single pathogen to studying the rhizome microbiome and how that community can interact to promote plant health via natural processes. Research is also highlighting the importance of microbiome interactions for contaminant degradation where the degraders are reliant on other organisms to provide vitamins or remove inhibitory compounds.

What do you think will be the next big topic in geomicrobiology research?

In my opinion, the next big topic in geomicrobiology is tackling the issue of waste and our increasing demand on critical minerals (metals and non-metals that are of high-value due to their economic importance and need in manufacturing high tech devices). Microbes are now known to degrade plastics and can control the fate of elements through redox cycling – harnessing these powers of microbiomes could help sustainability efforts and solve a number of anthropogenic issues. Solving the plastic waste issue is vitally important and finding ways to naturally promote microbial waste degradation is a hot topic. We also need to be thinking about how we can recover valuable materials, like critical minerals, from wastes. Legacy mining has resulted in waste rock piles that require management and mitigation, but they also contain critical minerals – studies are needed to understand how microbes could be used to recover these valuable resources and provide new economic opportunities.

What is your favourite bacterial taxa, and why?

It is impossible to pick just one! As a scientist interested in microbiomes, I interact with many different taxa as microbes are extremely diverse in soils and sediments. I do have favorite microbial metabolisms with anaerobes, acetylenotrophic (acetylene-degrading) microbes, and metal cycling bacteria being my top groups. If I had to pick just 3 taxa, I would go with *Geobacter, Thiomonas*, and *Syntrophotalea. Geobacter* are anaerobic bacteria that are well-known for their ability to reduce iron and uranium, which are processes I studied for my PhD. When I was involved in a project to name *Geobacter daltonii*, it felt like the culmination of all the skills I learned as a doctoral student — molecular biology, cultivation, geochemistry, and bioinformatics. Recently, we named new species of *Thiomonas* and *Syntrophotalea*. Work on our new *Thiomonas* isolates started back in 2008 with studies aimed at understanding how iron-oxidizing bacteria can affect heavy metal contaminants. It took several fantastic collaborations and the work of many graduate students to get the *Thiomonas* work completed and published. We recently named *Syntrophotalea acetylenivorans*, and our work with this organism led us to define the term “acetylenotroph” and hypothesize that acetylenotrophs plays an important role in contaminant degradation and nutrient cycling.

*This interview was conducted by Associate Editor George Inglis*.

